# Patterns of Outpatient Service Satisfaction among Low-Income Adults in Rural China: A Latent Class Analysis

**DOI:** 10.3390/healthcare10081380

**Published:** 2022-07-25

**Authors:** Peiyi Lu, Chunyu Yang, Jun Yao, Mingxia Xian, Mack Shelley

**Affiliations:** 1 Departments of Political Science and Statistics, Iowa State University, Ames, IA 50011, USA; peiyilu@iastate.edu (P.L.); mshelley@iastate.edu (M.S.); 2 College of Law and Political Science, Institute of Climate Change and Public Policy, Nanjing University of Information Science and Technology, Nanjing 210044, China; yangchunyuno.1@163.com; 3 School of Health Policy & Management, Institute of Healthy Jiangsu Development, Nanjing Medical University, Nanjing 211166, China; 4 School of Political Science and Public Administration, Soochow University, Suzhou 215123, China; mingxiaxian2022@163.com

**Keywords:** outpatient satisfaction, rural low-income adults, latent class analysis

## Abstract

(1) Background: Low-income rural residents in China are disadvantaged due to their financial vulnerability and insufficient access to resources, and this situation demands more research effort. This study examined the pattern of outpatient service satisfaction and its determinants among low-income adults in rural China. (2) Methods: Rural low-income respondents who used outpatient services in their local healthcare facilities in Jiangsu, China evaluated the access, cost, environment, doctor–patient interaction, and other topics during their outpatient visit (*N* = 662). Latent class analysis was used to identify the groups characterized by various dimensions of outpatient satisfaction. Multinomial logistic regression explored the determinants of class membership. (3) Results: Three latent classes were identified: 28.70% had low satisfaction, unsatisfied with every dimension; 20.69% reported medium satisfaction that valued doctor–patient relationships; and 50.60% had high satisfaction but thought that costs were high. Both low and medium satisfaction were associated with a higher proportion of self-paid fees. (4) Conclusions: Healthcare costs were an important determinant of outpatient service satisfaction. Medical social workers are suggested to be included in the medical team to help patients identify financial assistance. Special aid programs may be developed to help relieve rural low-income patients’ medical cost-related burden.

## 1. Introduction

Patient satisfaction is a commonly used outcome indicator in healthcare service quality research. Exploring the pattern of patient evaluations of their experiences in utilizing medical services can provide information for healthcare professionals and administrators about how to improve the users’ experiences and service management, which can help sustain patient loyalty [1]. Currently, most of the patient satisfaction theories are developed based on consumer satisfaction in market research, which emphasizes how healthcare services should meet the expectations of patients to achieve high levels of satisfaction [2]. Research has focused on how healthcare service quality determines the patients’ satisfaction [3]. Specifically, patient satisfaction is determined by many dimensions of healthcare service quality, such as interpersonal, technical, environmental, and administrative factors [4]. It is further shaped by individuals’ socio-demographic characteristics and contextual variables [5].

Numerous studies have examined patient satisfaction around the world, many of which were concerned with Europe [6]. Applications to China are different from studies in Western countries, considering China’s large population base and ongoing healthcare system reform. Violence against doctors in recent years further complicated the determinants of patient satisfaction in China [7]. More research on patient dis/satisfaction is warranted [8]. A nationwide investigation found that patient satisfaction in China should consider service delivery, doctor–patient communication/relationship, cost, treatment process, environment, and waiting time [9].

Prior research has called for more focus on rural China, where resources are relatively scarce, due to the underdeveloped economy in rural areas [10,11,12]. Compared to urban areas, the rural areas had lower healthcare expenditure from the government and thus less service access [13,14]. The rural low-income individuals are particularly disadvantaged regarding their health and financial status. People with a low income are at a higher risk of active diseases, physical impairment, functional limitation, and disability than their high-income counterparts [15]. Moreover, they have inadequate access to healthcare because of their financial vulnerability and the limited healthcare services available to them [13,16]. Studies have found that rural low-income individuals worry about the price when choosing healthcare providers [17], are less likely to use medical service [16,17], and are more likely to drop out from medical treatment [18]. Therefore, a better understanding of the healthcare use experience and its determinants is of great importance for policy or clinical practice to improve healthcare access and quality for this vulnerable population group. However, while some studies have focused on patient service satisfaction in rural China [10,12,19,20], few studies focused specifically on the rural low-income individuals.

Using the data collected in rural low-income households in Jiangsu, China, this study sought to classify rural low-income adults into several homogeneous groups, based on their outpatient service satisfaction and to explore the pattern of individuals’ responses to outpatient satisfaction items, using the latent class analysis approach. Based on class membership, we further examined the determinants of outpatient satisfaction class membership. The findings can help identify the pattern and determinants of outpatient satisfaction among the rural low-income residents, thereby providing implications for healthcare professionals to improve service quality and management. In particular, we would expect to identify a subgroup with low outpatient satisfaction and its risk factors, which could inform the development of an effective intervention strategy to improve medical use experience. These efforts to improve outpatient experience have the potential to benefit clinical effectiveness (e.g., adherence to medical instructions), and eventually improve patient safety and health outcomes [21,22].

## 2. Methods

### 2.1. Data and Sample

The data were used from a household study conducted in rural areas in Jiangsu province, China, in June, 2019. The list of counties with a disadvantaged economic status from 11 cities was obtained from the Jiangsu Commission of Health, which defined low-income areas as those where the per capita disposable income was below 6% of all rural households. In 2016–2020, the threshold was set to be less than CNY 6000 in per capita disposable income or less than CNY 180,000 per village. Some of the additional adjustments were applied to the South and Central areas of Jiangsu Province, based on the level of local economic development [23]. In general, only rural households whose per capita disposable income was below CNY 6000 were eligible for this study. A total of 58 counties were included in the investigation. In each county, at least two villages were randomly selected. In each village, we again obtained a list of low-income households, defined by the committee of the rural village, which is a quasi-government organization that implements policies and enforces local activities in rural villages in China. Finally, 10 low-income households were randomly chosen from each village. Many of these households became poor because at least one family member suffered from major illness and subsequent high medical fees. Only individuals aged at least 18 living in the chosen low-income households were eligible to participate.

The investigators were trained graduate students at the School of Health Policy and Management, Nanjing Medical University. The investigators visited the homes of the low-income households and conducted face-to-face interviews, accompanied by local government officers. A total of 1160 questionnaires were distributed and all of them were retrieved, for a 100% response rate. Among a total of 1155 valid adult respondents (99.57% effective rate), 662 used the outpatient service in their local healthcare facilities in the past month. Individuals who did not have an outpatient visit experience were not eligible to answer the questions about outpatient satisfaction and thus were excluded from this study. Therefore, this study used only 662 respondents as the analytic sample.

### 2.2. Measures

The demographic measures included age, gender, education, and marital status. Age was a continuous variable in years. Gender had two categories: female and male. Education was an ordinal variable with four levels: 1 = illiterate; 2 = primary school; 3 = middle school; 4 = high school and above. Marital status had three categories: unmarried; married; and widowed/divorced/separated.

The financial status covariates were individual income, Dibao eligibility status, and years in Dibao assistance. Income was an ordinal variable measuring an individual’s annual income: 1 = no income or in debt; 2 = CNY 0~6000; 3 = CNY > 6000. Dibao eligibility status indicated whether the household was eligible to receive Dibao, a government-funded monthly cash assistance for rural residents. The years in Dibao measured the time the household had received Dibao assistance.

The health measures and characteristics of the outpatient visit were included as covariates. The activities of daily life (ADL) measured the individuals’ difficulty to perform six daily life tasks (i.e., eating, bathing, dressing, toileting, indoor transferring, and urinary incontinence) (no = 0, yes = 1). Summing up all of the items provides a total score of ADL ranging from 0 to 6. A value of 0 indicated that the respondent was independent, while a value of 6 indicated that the person was dependent. The respondents were also asked if they had been diagnosed with any major or chronic illness (yes/no). The amount of the medical fee (unit: CNY 100) and the proportion of self-pay fees during the outpatient visit were recorded (range 0–1). Finally, the level of the healthcare facility was included: 1 = village clinic; 2 = town hospital; 3 = county hospital. Health insurance was not included as a covariate in this study because almost all of the respondents (99.7%) had health insurance. The lack of variability in the health insurance measure means that it would make no contribution to statistical model-fitting.

There are eleven indicators for outpatient satisfaction, based on well-established scales in previous studies ^13^. The respondents were asked to evaluate their experience of the most recent outpatient visit, including: (1) their time spent waiting; (2) the environment of the clinic; (3) if the diagnosis/treatment was understandable; (4) the extent of seriousness when doctors listened to their narratives; (5) whether the doctors showed respect when they explained their feelings; (6) the time doctors spent on treatment; (7) level of trust in doctors; (8) cost; (9) service quality; (10) convenience; and (11) overall satisfaction. All of the indicators were ordinal variables with three levels: 1 = poor; 2 = fair; and 3 = good.

### 2.3. Analytical Strategy

A descriptive analysis was first conducted to demonstrate the characteristics of respondents. Next, we used latent class analysis (LCA) to investigate the underlying pattern of outpatient satisfaction. The LCA is a popular statistical approach to identify latent categorical subgroups from a heterogeneous population with respect to a set of observed variables. This method is person-centered [24,25] and recommended to be used to examine patient satisfaction, because of its advantages of parsimony, connections with item response theory models, and ability to facilitate subsequent data analysis after identifying the latent classes [26]. There have been many applications in studying patient satisfaction [24,27], as well as job satisfaction among doctors [25,28].

The LCA was performed to identify the number of groups characterized by individuals’ responses to 11 outpatient satisfaction items without any covariates. Some goodness-of-fit indices were used to help determine the optimal number of classes, such as Bayesian Information Criterion (BIC), Akaike information criterion (AIC), adjusted BIC (ABIC), consistent AIC (CAIC), entropy (a pseudo *R^2^*), likelihood ratio test, etc. However, this study did not rely solely on the statistical criteria to choose the best model, but determined which model yielded meaningful interpretations when subdividing the population [25,29].

After deciding the number of classes, a new class membership of outpatient satisfaction was created for each respondent. Finally, multinomial logistic regression models were fit to examine the determinants of the class membership. The LCA was conducted in R using the package “poLCA” [30]. Multinomial logistic regression modeling was conducted in STATA.

## 3. Results

Table 1 shows the respondents’ socio-demographics. Many were older adults with a mean age of 62.46 years and a standard deviation (SD) of 13 years. Most of the respondents had attained education lower than high school, were married, and reported very low annual income. Half of them were eligible for Dibao assistance. About one-third had a major illness, although they were independent regarding ADL. For the outpatient visit, many of the respondents visited the village clinic and paid CNY 30. The proportion of self-pay was about 27%. The correlation matrix in Table 2 indicates that the continuous/ordinal demographic, financial, and health measures were weakly correlated. The highest correlation was between the amount of the medical fee and the levels of healthcare facility (*r* = 0.48).

In Table 3, we present the frequency distributions of all of the satisfaction indicators. Over 42% of the respondents provided a rating of good for most of the indicators, except time of treating and cost. In contrast, about 62% considered the time of treating as fair or poor, and 75% were unsatisfied with the medical cost.

Table 4 shows the model-fitting indices for the models with different numbers of classes. The model with three classes had the smallest BIC and CAIC and second-largest entropy. However, it did not achieve the smallest value in ABIC or AIC, and the LRT results indicated a model with more classes could be superior due to their larger likelihood. Considering BIC is the most reliable indicator of the true number of classes [30], and the three-class model had easier interpretability and more parsimony than a model with more classes; therefore, this study considered the three-class model to be the most appropriate solution.

Table 5 indicates the conditional probability of 11 satisfaction indicators rating “good” in the three-class model. Class 1 is defined as low satisfaction, with respondents unsatisfied with every dimension. This group reported the lowest probability in every dimension of outpatient satisfaction (<0.20) and accounted for 28.70% (*n* = 190) of the respondents. Class 2 is defined as medium satisfaction that valued the doctor–patient relationship. Many indicators in class 2 had a probability lower than 0.5, except for seriousness (0.73), show respect (0.75), and trust in doctor (0.61), indicating that this group highly valued the doctor–patient communication in the treatment process. It included 20.69% (*n* = 137) of respondents. Class 3 is defined as high satisfaction but thought costs were high. This group generally reported high satisfaction across all of the dimensions (>0.6), except cost (0.43). There were 335 respondents in this class (50.60%).

Multinomial logistic regression models were fitted to examine the predictors of new class memberships (Table 6). Low-satisfaction respondents were more likely to be male, have low income, and have a higher proportion of self-pay fees (relative risk ratios (RRR) < 1, *p* < 0.05) compared to the highly satisfied group. For the group with medium satisfaction that valued the doctor–patient relationship, the respondents were more likely to have higher medical fees and a higher proportion of self-pay fees compared to the high satisfaction group. In particular, a higher proportion of self-pay fees was associated with a greater risk of reporting low/medium satisfaction in both of the models (RRR > 1, *p* < 0.01).

## 4. Discussion

### 4.1. Summary of Key Findings

Using the first-hand data collected in rural Jiangsu, this study provides evidence of patterns of outpatient satisfaction among low-income individuals in rural China. The results illustrated three latent groups: low satisfaction unsatisfied with every dimension (28.70%); medium satisfaction that valued doctor–patient relationships (20.69%); and high satisfaction but thought costs were high (50.60%). The finding that about 29% of the respondents had low satisfaction highlighted that more efforts from China’s hospital and healthcare system are needed to improve their service quality.

In addition, this study examined the risk factors of low/medium outpatient satisfaction. The results revealed that economic factors, including individual income, amount of medical fee, and proportion of self-pay fees were strong determinants of outpatient satisfaction. These finding were consistent with previous studies on the determinants of patient satisfaction in China, again emphasizing the decisive effect of the patient’s economic affordability on their perceptions towards medical service quality [10,31]. Therefore, the focus should be on reducing medical costs for low-income individuals to improve outpatient service satisfaction. Specifically, considering the financial difficulties that low-income households may have, reducing the out-of-pocket cost can undoubtedly relieve their financial stress and subsequently improve the affordability of their healthcare.

Our results highlighted that the rural low-income outpatients care most about medical expenses when evaluating their satisfaction with the medical service. We postulate this finding in a broader sociopolitical context. In this study, most of the rural low-income respondents became poor due to major illness. While they have needs for medical treatment, their unfavorable financial ability cannot catch up with the accumulating high medical costs [7]. In the meanwhile, China’s healthcare policy reform has been struggling to address the issue of healthcare affordability [32]. To reduce the rural–urban healthcare inequality, the Chinese government has integrated the medical insurance systems for urban and rural residents. The new rural medical insurance scheme in China increased outpatient service use, but it did not significantly reduce the out-of-pocket costs for the rural residents [33]. For the rural low-income households, their financial burden relating to medical use remains heavy [34]. As a result, rural low-income outpatients still attach high importance to medical expenses, which is a reflection of the unaffordable care in China’s current healthcare system.

### 4.2. Implications for Policy, Practice, and Research

Some implications can be drawn for hospital administrators and healthcare policy-makers to improve outpatient satisfaction. The hospitals may develop a division of social service and hire social workers. The medical social workers could provide a case work service by helping patients identify financial assistance and matching them with other resources. The social workers’ service can relieve patients’ financial concerns, which ultimately can help improve patient satisfaction and service efficiency [35]. The healthcare affordability as a key determinant of the low-satisfaction group also points out the importance of relieving the cost-related pressure for rural low-income residents. Healthcare policy-makers should improve the current reimbursement rate for outpatient services. Special financial assistance programs may be developed for the rural low-income adults to ensure they have adequate access to medical services. For example, the local government may consider calibrating the out-of-pocket cost rates based on the income level of outpatients, or provide additional aids to patients experiencing financial difficulty.

### 4.3. Limitations

The above findings should be understood in light of some limitations. First, we focused only on rural low-income Chinese adults who used outpatient services. The limited sample representativeness prevented our findings from being extrapolated to other populations, inpatient service satisfaction, etc. A future study may consider including patients from urban areas or medium/high-income people, and conducting comparisons. In addition, this study included only the characteristics of patient demographics and outpatient visits as the predictors. Some of the factors, such as the type of outpatient service and the features of the hospitals and doctors, were not examined and could be worthy of future investigations. Finally, unlike some previous studies that sampled respondents from the hospital setting [36], the respondents of this study were a subset of a household survey sample. The sampling procedure in the community setting may leave out some eligible outpatients. The cross-sectional nature of this study also limited our ability to examine the changing features of outpatient satisfaction or to determine causal relationships.

## 5. Conclusions

Low-income rural people are at double jeopardy in accessing healthcare services, due to their disadvantaged financial status and the scarce-resource living environment. This study explored the pattern of their satisfaction during outpatient visits. The results indicated about 29% of the respondents had low satisfaction. The model results further highlighted out-of-pocket costs as an important predictor of outpatient satisfaction. It is suggested that additional policy to reduce the outpatient costs of rural low-income resident is needed.

## Figures and Tables

**Table 1:** Descriptive characteristics of the respondents, *N* = 662.

Variable	Mean ± SD/Median (Skewness)	*N* (%)	Variable	Mean ± SD/Median (Skewness)	*N* (%)
Age range (19–92)	62.46 ± 13.00		Dibao eligibility status—yes		335 (50.60)
Gender—male		406 (61.33)	Dibao eligibility status—no		325 (49.09)
Gender—female		256 (38.67)	Dibao eligibility status—missing		2 (0.30)
Education—illiterate		246 (37.16)	Years in Dibao assistance range (0–25)	0.33 (3.02)	
Education—primary school		232 (35.04)	ADL range (0–6)	0 (2)	
Education—middle school		151 (22.81)	Major illness—yes		216 (32.63)
Education—high school and above		33 (4.98)	Major illness—no		440 (66.47)
Marital status—unmarried		87 (13.14)	Major illness—missing		6 (0.91)
Marital status—married		425 (64.20)	Amount of medical fee range (0–70)	0.30 (4.15)	
Marital status—widowed/divorced/separated		148 (22.36)	Proportion of self-pay fee	0.27 (0.72)	
Marital status—missing		2 (0.30)	Level of healthcare facility—village clinic		437 (66.01)
Income—no income/in debt		303 (45.77)	Level of healthcare facility—town hospital		129 (19.49)
Income—CNY 0~6000		255 (38.52)	Level of healthcare facility—county hospital		96 (14.50)
Income—CNY > 6000		104 (15.71)			

Note: when the distribution of the variable was highly skewed, median and skewness were used to describe.

**Table 2:** Correlations between continuous/ordinal demographic, financial, and health measures.

	Age	Education	Income	Years of Poverty	ADL	Amount of Medical Fee	Proportion of Self-Pay Fee	Level of Healthcare Facility
Age	1							
Education	−0.29 ***	1						
Income	−0.08 *	0.03	1					
Years of poverty	0.03	0.02	−0.08 *	1				
ADL	−0.04	−0.03	−0.11 **	0.07	1			
Amount of medical fee	−0.09 *	0.08 *	−0.09 *	−0.06	0.14 ***	1		
Proportion of self-pay fee	0.01	0.02	0.08 *	−0.03	−0.02	0.15 ***	1	
Level of healthcare facility	−0.18 ***	0.08 *	−0.13 ***	−0.05	0.09 *	0.48 ***	0.19 ***	1

Note: * *p* < 0.05; ** *p* < 0.01; *** *p* < 0.001.

**Table 3:** Frequency distribution of 11 satisfaction indicators, *N* = 662.

Indicators	Poor *n* (%)	Fair *n* (%)	Good *n* (%)
Time waiting	124 (18.73)	259 (39.12)	279 (42.15)
Environment	43 (6.50)	279 (42.15)	340 (51.36)
Understandable	17 (2.57)	246 (37.16)	399 (60.27)
Seriousness	5 (0.76)	226 (34.14)	431 (65.11)
Show respect	7 (1.06)	214 (32.33)	441 (66.62)
Time treating	199 (30.06)	207 (31.27)	256 (38.67)
Trust in doctors	11 (1.66)	229 (34.59)	422 (63.75)
Cost	99 (14.95)	396 (59.82)	167 (25.23)
Service quality	25 (3.78)	249 (37.61)	388 (58.61)
Convenience	38 (5.74)	260 (39.27)	364 (54.98)
Overall satisfaction	15 (2.27)	268 (40.48)	379 (57.25)

**Table 4:** Model fit for the optimal number of classes characterized by outpatient satisfaction.

Model	Log-Likelihood	Resid. *df*	BIC	ABIC	AIC	CAIC	Entropy
Class 1	−6125.09	640	12,393.08	12,323.23	12,294.18	12,415.08	-
Class 2	−4638.71	617	9569.71	9426.83	9367.42	9614.71	0.96
**Class 3**	**−4486.68**	**594**	**9415.04**	**9199.14**	**9109.365**	**9483.04**	**0.88**
Class 4	−4416.67	571	9424.41	9135.48	9015.34	9515.41	0.83
Class 5	−4338.59	548	9417.64	9055.69	8905.18	9531.64	0.85

Note: Resid. *df* = degrees of freedom of residuals; BIC = Bayesian Information Criterion; AIC = Akaike information criterion; ABIC = adjusted BIC, CAIC = consistent AIC. The 3-class model in bold had the smallest BIC value.

**Table 5:** Conditional probability of “good” patient satisfaction indicators in the 3-class model.

Indicators	Class 1 (*n* = 190, 28.70%)	Class 2 (*n* = 137, 20.69%)	Class 3 (*n* = 335, 50.60%)
	low satisfaction	medium satisfaction	high satisfaction
Time waiting	0.07	0.39	0.63
Environment	0.05	0.41	0.83
Understandable	0.06	0.50	0.96
Seriousness	0.00	0.73	0.99
Show respect	0.05	0.75	0.99
Time treating	0.01	0.29	0.64
Trust in doctors	0.06	0.61	0.98
Cost	0.04	0.13	0.43
Service quality	0.06	0.45	0.95
Convenience	0.09	0.35	0.90
Overall satisfaction	0.19	0.33	0.90

**Table 6:** Multinomial logistic regression models results predicting outpatient satisfaction class membership.

	Class 1 vs. Class 3		Class 2 vs. Class 3	
	Low vs. High Satisfaction		Medium vs. High Satisfaction	
	RRR	95%CI	RRR	95%CI
Age	0.98	(96, 1.00)	0.99	(0.97, 1.00)
Gender—female	0.56 **	(0.37, 0.86)	0.72	(0.45, 1.15)
Education	0.85	(0.64, 1.14)	0.99	(0.76, 1.28)
Marital status—married	1.50	(0.91, 2.49)	0.83	(0.38, 1.82)
Marital status—widowed/divorced/separated	1.51	(0.71, 3.23)	0.94	(0.40, 2.18)
Income	0.61 **	(0.44, 0.84)	0.94	(0.69, 1.29)
Dibao eligibility status—no	1.41	(0.83, 2.41)	0.60	(0.31, 1.14)
Years in Dibao assistance	0.94	(0.85, 1.05)	0.85	(0.71, 1.01)
ADL	1.04	(0.91, 1.18)	1.02	(0.88, 1.18)
Major illness—no	0.67	(0.44, 1.03)	0.77	(0.49, 1.20)
Amount of medical fee	1.01	(0.98, 1.05)	1.04 *	(1.00, 1.07)
Proportion of self-pay fees	4.31 ***	(1.95, 9.53)	2.74 **	(1.41, 5.33)
Level of healthcare facility—town hospital	1.32	(0.74, 2.35)	1.60	(0.97, 2.66)
Level of healthcare facility—county hospital	1.65	(0.70, 3.89)	2.05	(0.95, 4.43)

Note: RRR = relative risk ratios; CI = confidence interval. Class 3 high satisfaction group was the reference group. The reference level for categorical variables: male for gender; unmarried for marital status; yes for Dibao eligibility status; yes for major illness; village clinic for level of healthcare facility. The standard errors were estimated by the bootstrapping method. Log-likelihood = −615.74, Wald Chi-square (30) = 130.55, Pseudo *R*^2^ = 0.0790. * *p* < 0.05; ** *p* < 0.01; *** *p* < 0.001.

## Data Availability

The datasets generated during the current study are available from the corresponding author on reasonable request.

## References

[1] Fatima T., Malik S.A., Shabbir A. (2018). Hospital Healthcare Service Quality, Patient Satisfaction and Loyalty. Int. J. Qual. Reliab. Manag..

[2] Batbaatar E., Dorjdagva J., Luvsannyam A., Amenta P. (2015). Conceptualisation of Patient Satisfaction: A Systematic Narrative Literature Review. Perspect. Public Health.

[3] Batbaatar E., Dorjdagva J., Luvsannyam A., Savino M.M., Amenta P. (2017). Determinants of Patient Satisfaction: A Systematic Review. Perspect. Public Health.

[4] Dagger T.S., Sweeney J.C., Johnson L.W. (2007). A Hierarchical Model of Health Service Quality: Scale Development and Investigation of an Integrated Model. J. Serv. Res..

[5] Naidu A. (2009). Factors Affecting Patient Satisfaction and Healthcare Quality. Int. J. Health Care Qual. Assur..

[6] Detollenaere J., Hanssens L., Schäfer W., Willems S. (2018). Can You Recommend Me a Good GP? Describing Social Differences in Patient Satisfaction within 31 Countries. Int. J. Qual. Heal. Care.

[7] Hesketh T., Wu D., Mao L., Ma N. (2012). Violence against Doctors in China. BMJ.

[8] Pan J., Liu D., Ali S. (2015). Patient Dissatisfaction in China: What Matters. Soc. Sci. Med..

[9] Tang L. (2012). The Influences of Patient’s Satisfaction with Medical Service Delivery, Assessment of Medical Service, and Trust in Health Delivery System on Patient’s Life Satisfaction in China. Health Qual. Life Outcomes.

[10] He X., Li L., Bian Y. (2018). Satisfaction Survey among Primary Health Care Outpatients in the Backward Region: An Empirical Study from Rural Western China. Patient Prefer. Adherence.

[11] Yan Z., Wan D., Li L. (2011). Patient Satisfaction in Two Chinese Provinces: Rural and Urban Differences. Int. J. Qual. Heal. Care.

[12] Gu D., Yang X., Li X., Liang C., Zhong J., Feng N. (2018). Innovating New Rural Cooperative Medical Scheme (NCMS) for Better Patient Satisfaction in Rural China. Int. J. Environ. Res. Public Health.

[13] Wang L., Wang A., Zhou D., FitzGerald G., Ye D., Jiang Q. (2016). An Empirical Analysis of Rural-Urban Differences in out-of-Pocket Health Expenditures in a Low-Income Society of China. PLoS ONE.

[14] Ying M., Wang S., Bai C., Li Y. (2020). Rural-Urban Differences in Health Outcomes, Healthcare Use, and Expenditures among Older Adults under Universal Health Insurance in China. PLoS ONE.

[15] Kumar K., Shukla A., Singh A., Ram F., Kowal P. (2016). Association between Wealth and Health among Older Adults in Rural China and India. J. Econ. Ageing.

[16] Lu P., Yang C., Yao J., Shelley M. (2020). Outpatient and Inpatient Service Use by Chinese Adults Living in Rural Low-Income Households. Soc. Work Public Health.

[17] Qian D., Pong R.W., Yin A., Nagarajan K.V., Meng Q. (2009). Determinants of Health Care Demand in Poor, Rural China: The Case of Gansu Province. Health Policy Plan..

[18] Jian W., Chan K.Y., Reidpath D.D., Xu L. (2010). China’s Rural-Urban Care Gap Shrank for Chronic Disease Patients, but Inequities Persist. Health Aff..

[19] Liu J., Mao Y. (2019). Patient Satisfaction with Rural Medical Services: A Cross-Sectional Survey in 11 Western Provinces in China. Int. J. Environ. Res. Public Health.

[20] Wang W., Maitland E., Nicholas S., Haggerty J. (2019). Determinants of Overall Satisfaction with Public Clinics in Rural China: Interpersonal Care Quality and Treatment Outcome. Int. J. Environ. Res. Public Health.

[21] Doyle C., Lennox L., Bell D. (2013). A Systematic Review of Evidence on the Links between Patient Experience and Clinical Safety and Effectiveness. BMJ Open.

[22] Price R.A., Elliott M.N., Zaslavsky A.M., Hays R.D., Lehrman W.G., Rybowski L., Edgman-Levitan S., Cleary P.D. (2014). Examining the Role of Patient Experience Surveys in Measuring Health Care Quality. Med. Care Res. Rev..

[23] Li X., Yang X., Li Y. (2019). The Differences of Relative Poverty among Rural Villages between Three Areas of Jiangsu Province. J. West..

[24] Chiou S.-J., Lee P.-C., Chang Y.-H., Huang P.-S., Lee L.-H., Lin K.-C. (2019). Assessment of Patient Experience Profiles and Satisfaction with Expectations of Treatment Effects by Using Latent Class Analysis Based on a National Patient Experience Survey in Taiwan. BMJ Open.

[25] Joyce C., Wang W.C. (2015). Job Satisfaction among Australian Doctors: The Use of Latent Class Analysis. J. Health Serv. Res. Policy.

[26] Cavrini G., Galimberti G., Soffritti G. (2009). Evaluating Patient Satisfaction through Latent Class Factor Analysis. Health Place.

[27] Frick U., Wiedermann W., Gutzwiller F.S. (2012). A Questionnaire on Treatment Satisfaction and Disease Specific Knowledge among Patients with Acute Coronary Syndrome. I: Are Treatment Satisfaction and Disease Specific Knowledge Continuous Latent Traits?. Patient Educ. Couns..

[28] Wang H., Tang C., Zhao S., Meng Q., Liu X. (2017). Job Satisfaction among Health-Care Staff in Township Health Centers in Rural China: Results from a Latent Class Analysis. Int. J. Environ. Res. Public Health.

[29] Lu P., Kong D., Shelley M., Davitt J.K. (2021). Intersectional Discrimination Attributions and Health Outcomes among American Older Adults: A Latent Class Analysis. Int. J. Aging Hum. Dev..

[30] Linzer D.A., Lewis J.B. (2011). PoLCA: An R Package for Polytomous Variable Latent Class Analysis. J. Stat. Softw..

[31] Wu J., Zhang S., Chen H., Lin Y., Dong X., Yin X., Lu Z., Cao S. (2016). Patient Satisfaction with Community Health Service Centers as Gatekeepers and the Influencing Factors: A Cross-Sectional Study in Shenzhen, China. PLoS ONE.

[32] Xiong C., Chen X., Zhao X., Liu C. (2018). Patient Satisfaction and Gender Composition of Physicians-a Cross-Sectional Study of Community Health Services in Hubei, China. BMC Health Serv. Res..

[33] Wagstaff A., Lindelow M., Jun G., Ling X., Juncheng Q. (2009). Extending Health Insurance to the Rural Population: An Impact Evaluation of China’s New Cooperative Medical Scheme. J. Health Econ..

[34] Guo N., Iversen T., Lu M., Wang J., Shi L. (2016). Does the New Cooperative Medical Scheme Reduce Inequality in Catastrophic Health Expenditure in Rural China?. BMC Health Serv. Res..

[35] Bristow D.P., Herrick C.A. (2002). Emergency Department Case Management: The Dyad Team of Nurse Case Manager and Social Worker Improve Discharge Planning and Patient and Staff Satisfaction While Decreasing Inappropriate Admissions and Costs: A Literature Review. Prof. Case Manag..

[36] Sun J., Hu G., Ma J., Chen Y., Wu L., Liu Q., Hu J., Livoti C., Jiang Y., Liu Y. (2017). Consumer Satisfaction with Tertiary Healthcare in China: Findings from the 2015 China National Patient Survey. Int. J. Qual. Heal. Care.

